# Bridging Duchenne muscular dystrophy therapy from rhesus monkeys to patients

**DOI:** 10.1002/ctm2.70786

**Published:** 2026-08-22

**Authors:** Wenting Guo, Weizhi Ji, Yongchang Chen

**Affiliations:** ^1^ State Key Laboratory of Primate Biomedical Research Institute of Primate Translational Medicine, Kunming University of Science and Technology Kunming China; ^2^ Faculty of Life Science and Technology Kunming University of Science and Technology Kunming China; ^3^ Yunnan Key Laboratory of Primate Biomedical Research Kunming China; ^4^ Southwest United Graduate School Kunming China

## 1 A DISEASE‐RELEVANT NONHUMAN PRIMATE PLATFORM

Duchenne muscular dystrophy (DMD) remains a fatal, progressive neuromuscular disease despite major advances in multidisciplinary care and genetic therapies. Its therapeutic landscape is complicated by thousands of pathogenic DMD variants and by differences in age, disease stage, muscle involvement, fibrosis, and immunity, all of which may influence treatment response. Successful translation therefore requires more than the production of detectable dystrophin. It requires matching therapies to the appropriate patients and disease stages, restoring biologically meaningful dystrophin across skeletal, respiratory, and cardiac muscles, maintaining efficacy for years, and achieving an acceptable safety profile.

To address these interconnected challenges, we began by using CRISPR/Cas9 to disrupt the *DMD* gene in rhesus monkeys in 2015.^1^, ^2^ Although the initial founders were mosaic, they established the feasibility of modelling dystrophin deficiency in primates. Subsequently, we spent nearly a decade obtaining hemizygous DMD monkeys that developed progressive muscle degeneration, markedly elevated serum creatine kinase, fibrosis, and motor impairment resembling early human DMD.^3^ More recently, we shortened the generation of patient‐relevant DMD monkeys from approximately 6–7 years to less than 1 year by directly introducing exon 50 mutations within the major exon 45–55 mutational hotspot.^4^ These models are not intended for high‐throughput screening; their value lies in mechanistic studies and evaluating systemic delivery, tissue distribution, motor function, immunogenicity, and durability under clinically relevant conditions. Mechanism dissection revealed early immune activation, abnormal fibro‐adipogenic progenitor differentiation, TGF‐β‐independent fibrosis, and intrinsic muscle stem‐cell dysfunction, which imply important therapeutic implications.^3^ Our recent work with circular ADAR‐recruiting RNAs (circ‐arRNAs) represents a translational milestone, linking therapeutic development and NHP validation to early clinical application.^5^

## 2 COMPLEMENTARY STRATEGIES FOR RESTORING DYSTROPHIN FUNCTION

Using this platform, we have explored several complementary therapeutic routes. Our initial antisense circular RNA (AS‐circRNA) strategy used stable, genetically encoded circular RNAs to bind DMD pre‐mRNA and obstruct spliceosome recognition.^6^ Adeno‐associated virus (AAV)‐delivered AS‐circRNA induced exon 51 skipping, restored the reading frame and dystrophin expression, and improved muscle pathology and function in Dmd mice. This work established that circular RNAs could serve not only as protein‐coding molecules or microRNA sponges, but also as durable regulators of pre‐mRNA splicing.

We next developed a single‐cut DNA‐editing strategy for exon 50‐mutant DMD.^4^ A muscle‐tropic AAV delivered the compact Cas12i^Max^ nuclease together with a single guide RNA targeting exon 51, inducing exon skipping or reframing without requiring two simultaneous DNA cuts. In a 17‐month‐old DMD rhesus monkey, one systemic administration restored dystrophin, improved muscle pathology and motor performance, and produced benefits lasting at least 1.5 years without detected off‐target editing or treatment‐related safety signals. This study also showed that mutation‐matched DMD monkeys can be generated and used for therapeutic evaluation within a clinically relevant development timeline.

A complementary, mutation‐independent strategy is to activate endogenous full‐length utrophin, a functional dystrophin homologue.^7^ We developed MyoAAV‐UA, which packages a compact dCasMINI‐VPR transcriptional activator and dual guide RNAs into a single muscle‐tropic AAV.^8^ Systemic treatment increased sarcolemmal utrophin, improved skeletal muscle function, and slowed cardiac deterioration in Dmd mice, with benefits detectable for 6 months. In healthy cynomolgus monkeys, MyoAAV‐UA increased skeletal‐muscle utrophin expression by more than twofold without significant toxicity, while patient‐derived myotubes showed restoration of the utrophin‐glycoprotein complex. Because this approach does not depend on the location or type of DMD mutation, it could potentially extend therapeutic coverage beyond patients eligible for a specific exon‐skipping or editing strategy.

## 3 FROM MONKEYS TO PATIENTS

NHP models bridge the translational gap between preclinical therapeutic development and clinical application. In our recent work, we evaluated a circ‐arRNA‐guided exon‐skipping strategy in three mutation‐matched DMD rhesus monkeys.^5^ After one systemic administration, exon skipping was detected across multiple muscles, accompanied by sarcolemmal dystrophin restoration, reduced muscle‐injury biomarkers, improved histopathology, and better motor performance. Benefits persisted for 1 year, with motor improvement in the exon 50 model remaining evident for 1.5 years. The younger exon 5 model showed clearer functional recovery than the older model despite molecular correction in both, suggesting that treatment before extensive irreversible muscle loss may be more effective. Dystrophin‐reactive T‐cell signals emerged in the exon 5 model but not in the exon 50 model, further suggesting that immune risk may depend on which dystrophin epitopes are reintroduced.

This strategy could potentially apply to approximately 13% of patients with DMD. Under compassionate‐use approval, three ambulatory boys aged 4.6–6.6 years received a single intravenous infusion (LE051). Eight‐week muscle biopsies showed dose‐related exon skipping and dystrophin restoration. Improvements in North Star Ambulatory Assessment scores, timed motor tests, and 6‐minute walk distance were observed during follow‐up, together with exploratory cardiopulmonary signals in one participant. No treatment‐related serious adverse events or dose‐limiting toxicities were reported.^5^ Together, the concordant molecular and functional findings across DMD NHP models and patients support the translational potential of circ‐arRNA‐guided exon skipping, although larger controlled trials and longer follow‐up are required to establish its durability, long‐term safety, and clinical efficacy.

## 4 CLINICAL IMPLICATIONS AND FUTURE DIRECTIONS

Several implications are immediately relevant to clinicians. Precise genotyping is essential for determining exon‐skipping eligibility and assessing the potential reintroduction of immunogenic dystrophin epitopes. Early referral may also be important, because molecular correction cannot restore muscle already irreversibly lost to fibrosis. Clinical monitoring should extend beyond serum creatine kinase and ambulatory performance to include liver enzymes, platelet counts, AAV immunity, dystrophin‐specific cellular responses, cardiac imaging, pulmonary function, and, where appropriate, molecular confirmation in muscle. The early LE051 experience represents an important translational step, but controlled trials and long‐term follow‐up are now essential to determine whether these molecular and functional signals translate into durable clinical benefit.

**FIGURE 1:**
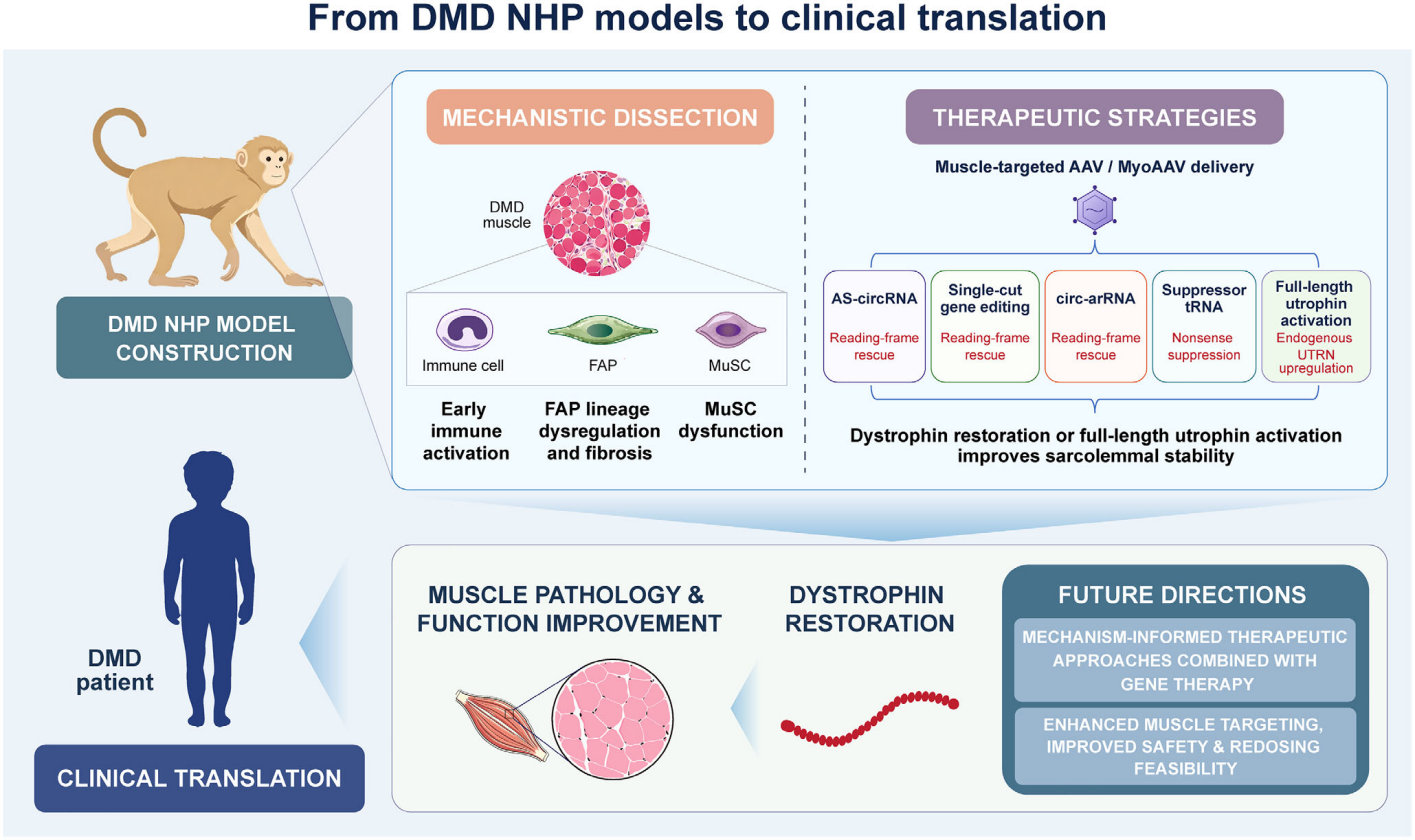
From Duchenne muscular dystrophy (DMD) NHP models to clinical translation. DMD rhesus monkey models enable mechanistic dissection as well as the development and evaluation of mutation‐matched therapeutic strategies, facilitating translation of candidate therapies for human DMD patients.

Our parallel development of single‐cut gene correction, AS‐circRNA‐mediated exon skipping, suppressor‐*t*RNA therapy,^9^ and mutation‐independent activation of endogenous full‐length utrophin provide a complementary, mutation‐informed therapeutic framework for DMD. With further preclinical validation and safety assessment, each of these strategies has the potential to advance into formal clinical trials. Treatment should be informed by genotype, disease stage, and potential immune risk. A shared bottleneck across these approaches is the broad and safe delivery of therapeutic cargo to skeletal, respiratory, and cardiac muscles. Future optimisation should further enhance muscle coverage, lower vector dose and hepatic exposure, and tackle AAV immunity as well as the redosing challenge. Moreover, mechanism‐informed therapeutic strategies should be integrated with gene therapy to enhance therapeutic efficacy. Within this framework, NHP models serve not merely to reproduce the disease, but to match therapies to patients, identify clinically relevant risks, and make first‐in‐human treatment more informed (Figure 1).

## CONFLICT OF INTEREST STATEMENT

The authors declare no conflict of interest.

## Data Availability

Data sharing not applicable to this article, as no datasets were generated or analysed during the current study.
